# Effect of LongRange™ eprinomectin on *Anopheles arabiensis* by feeding on calves treated with the drug

**DOI:** 10.1186/s12936-019-2964-y

**Published:** 2019-09-30

**Authors:** Aklilu Belay, Beyene Petros, Teshome Gebre-Michael, Meshesha Balkew

**Affiliations:** 1 Aklilu Lemma Institute of Pathobiology, Addis Ababa University, Addis Ababa, Ethiopia; 2 Department of Microbial, Cellular & Molecular Biology, College of Natural Sciences, Addis Ababa University, Addis Ababa, Ethiopia; 3 Abt Associates, PMI VectorLink Project in Ethiopia, Addis Ababa, Ethiopia

**Keywords:** *Anopheles arabiensis*, Cattle, LongRange™ (eprinomectin 5%), Malaria control, Ethiopia

## Abstract

**Background:**

Misuse of long-lasting insecticidal nets together with resistance of vectors to most of the insecticides for indoor residual spraying and impregnated nets threaten malaria vector control interventions, requiring search for alternative control methods. Reports have shown that *Anopheles* mosquitoes die when they feed on endectocidal drugs used to treat humans and animals. A study was designed to investigate the efficacy of LongRange™ (eprinomectin 5%) on laboratory reared *Anopheles arabiensis* fed on treated calves.

**Methods:**

*Anopheles arabiensis* from insectary colony was fed on three calves treated with therapeutic dose of LongRange™ eprinomectin (1 ml/50 kg) and on non-treated three other calves as control arm. For the feeding, mosquitoes were placed in paper cups covered with nylon cloth mesh and then allowed to feed on the necks of calves. Subsequently, mosquito survival, fecundity, egg hatchability, larval development and adult emergence were recorded. Data were entered and analysed by using SPSS version 20. The Kaplan–Meier survival analysis and independent sample t-test were used.

**Results:**

All mosquitoes that fed on LongRange™ Eprinomectin treated calves died within 7 days following blood ingestion. The drug also slightly affected fecundity and hatchability of *An. arabiensis*.

**Conclusion:**

Treating livestock with LongRange™ (eprinomectin 5%) may serve as a supplementary control method for zoophagic *An. arabiensis.*

## Background

In Ethiopia, malaria showed a declining trend over the last 15 years mainly due to the high coverage of key control interventions, such as artemisinin-based combination therapy (ACT), use of rapid diagnostic tests (RDTs) at the remote health facilities, wide-scale distribution of long-lasting insecticidal nets (LLINs) and increased coverage of indoor residual spraying (IRS) since 2004/2005 [1, 2]. As a result, malaria decreased from 4.1% in 2006 to 0.4% in 2007 [3]. A survey in 2012 showed reduction in malaria admissions by 54% and deaths by 55%, as compared to the rates in 2001–2004 [4]. Ethiopia also attained a reduction in malaria morbidity from 22 to 10%; malaria case fatality rate from 4.5 to 2% in age groups of 5 years and above, and 5% to 2% in under 5 children [4].

The vector control interventions targeted *Anopheles arabiensis* [5, 6], which is a member of the *Anopheles gambiae* complex [7] and the main vector of the disease in the country. *Anopheles pharoensis, Anopheles funestus* and *Anopheles nili* serve as secondary vectors in some areas [8–11].

Although the World Health Organization (WHO) recommends LLINs and IRS for malaria control in many African countries [12, 13], insecticide resistance appears to be a major challenge for malaria vector control programmes [14, 15]. The challenge became very serious with the occurrence of pyrethroid resistant *An. funestus* in southern Africa [13] and multiple insecticide resistant populations of *An. arabiensis* in some other countries [16]. Also in Ethiopia, high levels of resistance to DDT and the pyrethroid insecticides [10, 15, 17] were reported from the South, Gambella, Gorgora and the Ghibe River valley.

Furthermore, additional factors like improper use of LLINs and increased outdoor feeding frequency of malaria vectors may negatively affect the benefits of nets [18], implicating the need for novel approaches that will supplement the available vector control tools by reducing the use of conventional insecticides. This would reduce the rate of resistance development to the existing insecticides and also target vectors that feed and/or rest outdoors [19].

Endectocides, the macrocyclic lactones (avermectin/milbemycin), are drugs with endoparasitocidal and ectoparasitocidal activities [20]. They have been used to treat metazoan parasites from multiple phyla [21]. Treatment of livestock with endectocides has been reported to be effective against zoophilic malaria vectors [22], tsetse flies, and ticks in sub-Saharan Africa [23]. One of the endocticides is ivermectin, which is known to be toxic to mosquitoes [24] that feed on treated humans and cattle [25]. Studies conducted by Fritz and her colleagues [26, 27] showed that mass treatment of livestock with ivermectin could reduce zoophilic vector populations at the onset of the rainy season, precluding epidemics in malaria endemic regions. A therapeutic dose of ivermectin in a mosquito blood meal causes reduced survival, fecundity, and egg hatch rate [26, 28–31]. This has been shown to serve as a supplementary approach to prevent malaria transmission by reducing the population of *Anopheles* mosquitoes [31]. Its effect is enhanced as it affects the age structure of the vector population by changing/pulling it towards the younger age class which does not participate in transmission [32].

As with ivermectin, LongRange™ (eprinomectin 5%) has a deworming action in cattle and it persists in treated cattle for 150 days [33]. Hence, the present study investigates the efficacy of LongRange™ eprinomectin (LRE) against insectary-reared *An. arabiensis* in Ethiopia.

## Methods

### Study area

The study was conducted in Edo Kontola (a rural village) which is located about 4 to 5 km north of Batu town (Ziway) from February to May 2016. Batu town is located 167 km South of Addis Ababa, Ethiopia. The area lies at an average altitude of 1653 m above sea level. The total annual rainfall is approximately 700 mm, with peaks during the main rainy season in July (250 mm) and August (220 mm). The mean minimum and maximum annual temperatures are 14.5 °C and 27.7 °C, respectively.

### Study design

A randomized control trial was conducted on six calves—three control and three treatment. Epirnomectin was subcutaneously injected by a veterinarian and the efficacy of the drug on *An. arabiensis* was assessed for 85 days.

### Mosquitoes

A colony of *An. arabiensis* that was maintained at Aklilu Lemma Institute of Pathobiology, Addis Ababa University since 2001 was used in the study. The generation of these mosquitoes was F-165 (where F is for filial) and they were reared at 28–31 °C, 70–80% relative humidity and a 12:12 lightdark cycle. It was originally collected from Bishoftu (Debrezeit), which is located at 45 km East of Addis Ababa [10]. The colony is susceptible (100% mortality) to DDT, permethrin and deltamethrin [10]. Larvae were raised on a diet of ground Tetramin^®^ fish meal and adults maintained on 10% sterile sugar solution. A high bovine blood index of *An. arabiensis* in the village was known from the study of Gari et al. [34].

### Experimental drug

The LongRange™ (eprinomectin 5%), “LongRange™”, a trademark of Merial (registration pending in the USA) [33], was kindly provided by Dr. Seth Irish, research entomologist at CDC in Atlanta and stored as recommended by the manufacturer and used in the study. It is a semi-synthetic compound of the ivermectin family drugs and was originally selected as a nematocide, insecticide, and miticide [35, 36].

### Treatment of calves with eprinomectin

The experiment was performed following the method used by Naz et al. [22] with some modifications. Six calves (1–3 year old) of local breed having approximately equal weights were divided into two groups (three calves per group). Calves were kept together with the herd in the field grazing around Lake Ziway, 5 km North East of Batu town. The body mass of the calves was measured by tightly stretching the Heart Girth (mass measuring metre) [37] behind the fore limbs from the ventral to dorsal (hump) area. Prior to conducting the study, information on the history of previous drug exposure was checked for each calf, to avoid possible interference from other drugs that may affect mosquitoes 1 week ahead and a day before drug injection. The drug dose for each calf was calculated (1 mg/kg which is equivalent to 1 ml/50 kg) according to the instruction of the manufacturing company. The drug was injected to the calves by a veterinarian subcutaneously under the loose skin in front of the shoulder by using 18 gauge, ½ in. needle after sanitizing the area with denatured alcohol.

### Bioassay of eprinomectin against *Anopheles arabiensis* that fed on drug dosed calves

The bioassay was done according to Poche et al. [38], with little modification. Briefly, paper cups were 24 h after drug injection to the calves, 20 unfed *An. arabiensis* females 3–5 days old were transferred into paper cups covered with nylon netting by using mouth aspirator. A total of 40 *An. arabiensis* in two cups of 20 individuals were allowed to feed on the neck of each calf for one hour in the evening (7:00–8:00 p.m.) by wrapping the cups on the left and right sides of their neck with a stripe of cotton cloth. After the feeding session, fully engorged mosquitoes were carefully selected and transferred into small Barraud cages that were covered with a large polyethylene bag by using glass tube mouth aspirator. In the cages, the mosquitoes were provided with 10% sterile sugar solution soaked in cotton pads. Cotton pads soaked in water were also placed in the polythene bags to maintain 70–80% humidity. The controls and all blood-fed mosquitoes were maintained inside a thatched hut field laboratory, under natural temperature condition, and followed for mortality for four consecutive days. Mortality was recorded from each cage every 24 h. A correction for treatment response was made from control response by using the Abbott’s formula [39]. However, since the control response values in the present study were < 10% and had only small effect on the value of mean experimental treatment responses, the application of Abbott’s formula was not found applicable. The drug is claimed to provide a second peak concentration in the plasma after 70 days of a single dose treatment on calves [35]. Therefore, exposure of mosquitoes was conducted every week for about 85 days to treated and untreated calves.

### Observations on the effect of eprinomectin on the fecundity of *Anopheles arabiensis*

In addition to mortality, observations were carried out on the effect of eprinomectin on fecundity and fertility of *An. arabiensis.* Using the method described by Pooda et al. [40], mosquitoes surviving 4 days post-blood feeding on drug-dosed calves were grouped into two; half were dissected for fecundity (egg counting) and the remaining half were observed for egg hatchability. Ovarial dissection was done under a 40× dissecting microscope and the number of eggs from each mosquito counted and recorded.

### Observations on the effect of eprinomectin on *Anopheles arabiensis* egg hatchability

The fertility of mosquitoes was determined based on the method of Derua et al. [41]. This was done for each group (treatment and control) of mosquitoes. On weeks 2, 4 and 5, treatment group mosquitoes were placed in one cage and the control group mosquitoes in another cage and followed for egg oviposition for 4 days by providing egg laying substrates (wetted filter papers on petri dishes). Eggs were counted and then transferred to rearing pans for incubation and hatching. Upon hatching, all first instar larvae were counted and removed daily and this was done for 3 days [26]. Fertility of individual mosquitoes was monitored from 6 to 12 weeks post drug exposure, by placing them in a plastic cup covered with nylon cloth mesh, with a lining of wet filter paper for egg laying. Five mosquitoes from each cage (12 cages; a total of 60 mosquitoes) were transferred singly into 60 plastic cups. Eggs laid from each cup were counted and transferred into individual pans to maintain and follow larval development up to adult emergence during which the number of larvae, pupae and adults were counted and recorded.

### Data analysis

Data were entered into Microsoft Excel and analysed using IBM, SPSS version 20. Mortality data was compared between treatment and control arms using Kaplan–Meier estimator. The percent of fed mosquitoes were obtained by calculating the ratio of total fed to total exposed. The fecundity, fertility and larval development data was compared between the two groups using independent t-test. The comparison was based on means of the target variables, i.e., mortality, fecundity and fertility rates obtained from both treated and control groups. Statistical significance was assumed whenever P-values are < 0.05.

## Results

### Blood feeding and mortality rate of *Anopheles arabiensis*

A total of 3020 female *An. arabiensis* mosquitoes were exposed to feed on calves among which 1510 were eprinomectin-treated and the other 1510 untreated controls (Table 1). From the 1510 *An. arabiensis* that were allowed to feed on treated calves, 81.3% (1228/1510) were blood fed and while the control feeding rate was 83.2% (1256/1510).

**Table 1:** Blood feeding rates of mosquitoes exposed to LRE treated and untreated calves during 12 weeks experiment where mosquitoes were exposed for each test at Edo Kontola, Batu, Feb.–June, 2016

Week^a^	Host animal group	Mean ± SD number of fed mosquitoes (n = 120)	Percent fed mosquitoes	P-value
0	Treated	27 ± 6	67.50	0.800
	Control	26 ± 2^b^	78.0	
1	Treated	32 ± 1	80.0	0.442
	Control	33 ± 2.4	83.30	
2	Treated	27 ± 4	67.50	0.116
	Control	35 ± 2.6	87.50	
4	Treated	32 ± 5	80.0	0.847
	Control	33 ± 5	81.70	
5	Treated	35 ± 3	86.70	0.368
	Control	37 ± 2	91.70	
6	Treated	37 ± 5	93.30	0.651
	Control	36 ± 1	90.0	
7	Treated	38 ± 0.7	94.20	0.116
	Control	37 ± 0	92.50	
8	Treated	39 ± 0.7	96.70	0.101
	Control	38 ± 0.7	94.20	
9	Treated	35 ± 4	86.70	0.468
	Control	37 ± 2	91.70	
10	Treated	39 ± 1	97.50	0.279
	Control	37 ± 2	93.30	
11	Treated	33 ± 4	83.30	0.116
	Control	38 ± 2	95.80	
12	Treated	36 ± 3.6	90.0	0.138
	Control	32 ± 1	80.0	

*SD* standard deviation

^a^The week 3 trial data were disregarded because of death of mosquitoes in the control arm with an unknown reason

^b^The number exposed mosquitoes were 100

There was no significant difference in the mean number of *An. arabiensis* that fed on treated calves as compared to the corresponding controls in each week of feeding trial. From a total of 120 exposed mosquitoes per week, the mean feeding count of *An. arabiensis* on drug-treated calves was 102 (range: 81–117) and the control calves was 106 (range: 94–115) (Table 1). The fact that feeding of *An. arabiensis* with a similar preference on the two groups implies absence of repellent effect of eprinomectin treated calves on mosquitoes.

There were variable mortality rates of blood fed *An. arabiensis* between the trial weeks. Mortality of *An. arabiensis* fed on drug-treated calves was 100% on week 0 (day 1) and week 1 (day 7). All mosquitoes on week one died within 36 h post-feeding. On week two, mortality were reduced to 35% that deaths started after 2 days (48 h) of living post-feeding. In the first and second weeks, the respective control mortalities were 5% and 8%. The week 3 trial data were disregarded because of death of mosquitoes in the control arm with an unknown reason.

**Fig. 1:**
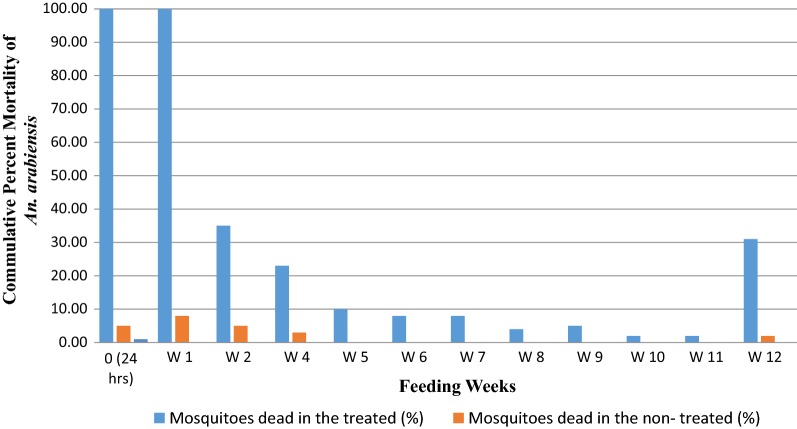
Mortality of blood fed *An. arabiensis* mosquitoes on calves following a single injection of LRE at Edo Kontola, Batu, Central Ethiopia, Feb., 2016–May, 2016

The differences in the mortality of *An. arabiensis* between treated and the corresponding control group were statistically significant on weeks 2 to week 7 (P ≤ 0.008) although the percent mortalities of these weeks were very low (ranges 35% to 8%). There was no difference between mortality of the treated and controls for weeks 8, 9, 10 and 11. However, mosquitoes that fed on day 85 showed significant difference in mortality (P < 0.001) (Fig. 1).

### Fecundity of *Anopheles arabiensis*

Among those *An. arabiensis* that fed on treated calves, 593 gravid were dissected and a total of 13,629 eggs were harvested (Table 2). Similarly, 573 gravid mosquitoes were dissected from the control and a total of 18,388 eggs were obtained. The mean oviposited egg count by those fed on treated calves was 23 (range: 9–37) eggs/per mosquito, while the corresponding mean number from the controls was 32 (range: 24–42) eggs per mosquito. The difference between egg counts of the two groups was not statistically significant.

**Table 2:** Fecundity of *Anopheles arabiensis* that fed on LRE treated calves, Edo Kontola, Batu, Central Ethiopa, Feb.–May 2016

Parameter	Weeks post injection									
	2	4	5	6	7	8	9	10	11	12
No. dissected in the treated	20	40	47	74	83	69	85	85	45	45
Mean no. of eggs ± SD/mosquito	35 ± 4	27 ± 6	38 ± 3.4	17 ± 2.6	33 ± 3.6	24 ± 3.6	20 ± 5	10 ± 3	21 ± 3	21 ± 6
No. dissected in the control	20	40	46	74	83	69	82	69	45	45
Mean no of eggs ± SD/mosquito	40 ± 2.6	31 ± 2.6	44 ± 3.6	25 ± 4.6	37 ± 2	27 ± 6	24 ± 7	26 ± 2.3	42 ± 2	42 ± 4
P-value	0.883	0.626	0.594	0.379	0.196	0.449	0.452	0.648	0.326	0.086

*SD* standard deviation

### Fertility (egg hatchability) of *Anopheles arabiensis*

**Fig. 2:**
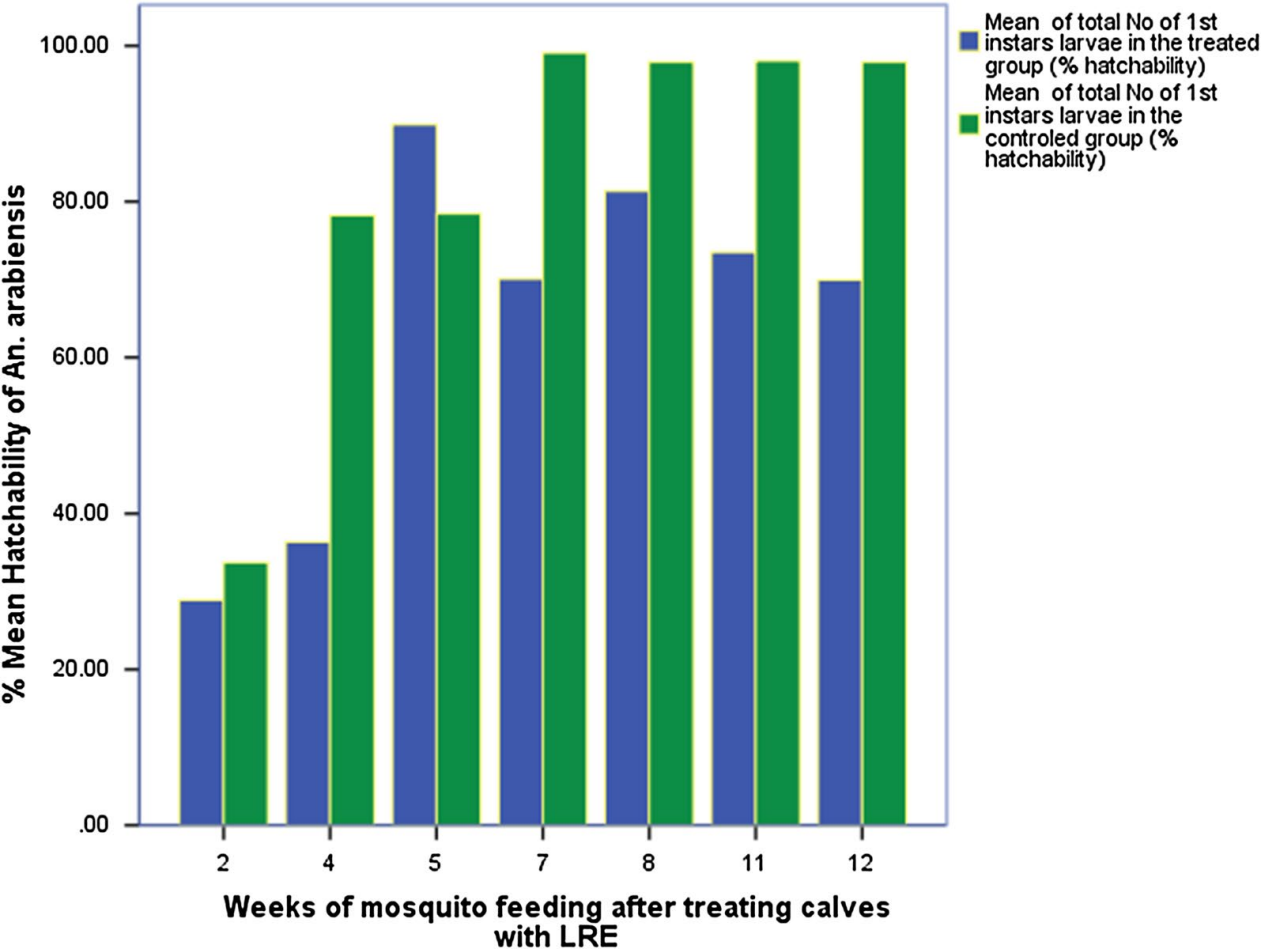
Comparison of percent hatchability of eggs laid by *An. arabiensis* mosquitoes that survive after feeding on treated calves versus controls. *The week 9 and week 10 fed did not lay eggs

A total of 466 gravid *An. arabiensis,* of which 233 that fed on treated cattle and 233 on controls were compared for egg laying (Fig. 2). A total of 2235 eggs were laid by the control group and 2227 by the mosquitoes that fed on treated calves. From treated group eggs, 1343 first instars (mean 64 ± 8.6 per mosquito) hatched with 60% hatchability, whereas 1771 first instars (mean 83 ± 8.9) with 79% hatchability hatched from the controls. Rate of hatchability of eggs of mosquitoes that fed on treated calves was 19% lower, compared to controls.

### Larval development and adult emergence

From a total of 1771 larvae in the control arm, 374 were transformed into pupae and from 1343 larvae in the treated group 316 pupae were obtained resulting in 21% and 24% pupation rate, respectively. In addition, the probability of adult emergence from pupae in the control group was 42% (6% to 95%) and in the treated group was 41% (4% to 70%). However, the drug significantly reduced the development of larvae and adult emergence on week 4 (P = 0.013), week 5 (P = 0.019) and week 11 (P < 0.001) trials (Table 3).

**Table 3:** Average number of pupae and adult emergence from eggs of *Anopheles arabiensis* that fed on LRE treated calves and controls, Batu, Central Ethiopia, Feb., 2016–May, 2016 (85 days)

Week	Animal group	Average no. of pupae ± SD	Percent pupation	Average ± SD no. of adult emergence	Percent adult emerged from larvae	P-value
2	Control	11.3 ± 1.15	63	11.3 ± 1.15	63	1
	Treated	11.3 ± 2.3	71	11.3 ± 2.3	71	
4	Control	28.3 ± 7.6	13	28.3 ± 7.6	13	0.013*
	Treated	9.7 ± 0.58	15	9.7 ± 0.58	15	
5	Control	12.3 ± 2.5	6	12.3 ± 2.5	6	0.019*
	Treated	6.7 ± 0.58	5	6.7 ± 0.58	4	
6	Control	22.3 ± 20.4	56	22.3 ± 20.4	53	0.663
	Treated	27.7 ± 25	47	27.7 ± 25	56	
7	Control	29.7 ± 22.8	65	29.7 ± 22.8	95	0.798
	Treated	24.3 ± 22	95	24.3 ± 22	65	
8	Control	7.3 ± 6.7	15	7.3 ± 6.7	17	0.362
	Treated	16 ± 13	13	16 ± 13	15	
11	Control	13.3 ± 0.58	85	13.3 ± 0.58	45	< 0.001*
	Treated	9.7 ± 0.58	31	9.7 ± 0.58	62	

*SD* standard deviation

* P-values are statistically significant

## Discussion

This research evaluated the endectocidal activity of eprinomectin in calves against *An. arabiensis* in a semi-field environment. The effects of the drug with varying degrees of efficacy were observed on the later feeding weeks from treatment. The finding that mortality of all the mosquitoes that fed on treated calves within a week of treatment shows that eprinomectin can reduce adult zoophagic *An. arabiensis* populations. A previous study in Kenya also reported that eprinomectin mixed with bovine blood and provided to laboratory-reared *An. arabiensis* in a membrane feeder killed all mosquitoes at low concentrations [27]. In the present study, the drug effect was significantly higher up to 7 weeks post-treatment. However, the rate of mortality of *An. arabiensis* after a week was significantly reduced. This finding is in agreement with previous studies by Poché et al. [36] in Western Kenya where eprinomectin, fipronil and ivermectin were found effective killing *An. arabiensis* for at least 7 days at the lower doses of 0.25 and 0.5 mg/kg and at a concentration of 0.2 mg/kg for up to 21 days post-treatment.

A similar study by Derua et al. [41] using ivermectin also showed that *An. gambiae* that fed on treated human volunteers were killed within 3 days post-treatment. The studies present evidences on the potential impact of endectocides including eprinomectin, on vector mosquitoes, and hence, it can be suggested that where the vectors are zoophagic and outdoor feeders combining cattle treatment with IRS and LLINs could be taken alternative approaches in vector control interventions particularly during epidemic seasons. The approach can also be a component of integrated vector management. In areas where there is continuous transmission, there could be a need for repeated treatment of cattle and humans with endectocides.

Eprinomectin was observed to be very effective in protecting cattle from a variety of nematode parasites [33, 35] which is an added advantage in countries like Ethiopia where malaria and other parasites of humans and animals are most common. In contrast with ivermectin, eprinomectin has a better pharmacokinetic profile with possibly longer effect and has no withdrawal period for milking i.e. a treated cow can be milked right after treatment and the milk for human consumption is safe as the drug has limited distribution in milk [33]. In addition, eprinomectin can provide high levels of parasite control against a range of nematodes of cattle for up to 5 months following a single treatment [35].

Thus, treatment of cattle with eprinomectin can be implemented as a supplementary control of *An. arabiensis* in conditions where the mosquito is zoophagic and exophagic, crepuscular and resistant to the available insecticide-based control methods, such as LLINs and IRS in particular and outdoor malaria transmission in general.

*Anopheles arabiensis* that fed on treated calves were observed to lay relatively lesser number of eggs (not statistically significant) but their reproduction capacity was not affected. This is similar with a study by Fritz et al. [27] which showed no difference in the fecundity of *An. arabiensis* fed on eprinomectin mixed bovine blood and DMSO-treated blood (control) in a membrane feeder system. The delay in hatching time, larval development, pupation and adult emergence detected in the present study in mosquitoes fed on treated calves than the non-treated is similar to that reported by Pooda and his colleagues, in which it was shown that a therapeutic dose of ivermectin delayed the first larviposition of *Glossina palpalis gambiensis* [30]. According to previous reports, factors like delay in the ovulation process, an increase in the duration of maturation, and/or a disruption of pupation reflect the effect of endectocides on the fertility of flies [42].

In the present study, the temperature fluctuation in the area might have affected the larval development in the field laboratory. This is consistent to the observation made by Beck-Johnson et al. [43] and others [44] who reported that the fall in mean temperature below 18 °C inhibits egg development. The data showed that such temperature changes affected larval development and adult emergence on weeks 4, 5, 8, 9 and 10. Yang et al. [45] also observed that, the increase in ambient temperature is a beneficial factor for the maintenance of vital physiological activities of mosquitoes leading to maturation of fertilized eggs.

The effective weekly test of mosquitoes, maintenance of fed laboratory, rearing and controlling the daily temperature and humidity at the field laboratory were the strengths of the present study. The limitations of the study were that, the exposed mosquitoes should have been wild-caught and the study should have run for up to 120 days to generate a more robust data, which unfortunately was not financially and technically possible.

## Conclusions

Treating calves with a therapeutic dose of eprinomectin killed *An. arabiensis* that fed on calves within 7 days post-treatment. However, mortality of *An. arabiensis* was reduced gradually after day seven post-treatment. The drug showed no significant effect on fecundity and fertility of *An. arabiensis*. Based on the present study, eprinomectin has the potential for use as a supplementary vector control tool together with IRS and LLINs, against *An. arabiensis,* if weekly mass drug administration of cattle is used for controlling zoophilic mosquitoes and reduce outdoor malaria transmission. This would fill the gap in mosquito control where the country is mainly using control measures targeting the human–vector contact, which would fail as mosquitoes shift their feeding behaviour from humans to domestic cattle. However, the present findings must be refined through future studies that are based on large sample size, use of tent traps and allowing wild caught mosquitoes to feed and by conducting the study under different eco-epidemiological conditions.

## Data Availability

Data is available whenever it is necessary.

## Abbreviations

LLINs: long-lasting insecticidal nets
ITNs: insecticide-treated nets
DDT: dichlorodiphenyltrichloroethane
IRS: indoor residual spray
ACT: artemisinin-based combination therapy
RDTs: rapid diagnostic tests
WHO: World Health Organization
ALIPB: Aklilu Lemma Institute of Pathobiology
